# Risk-stratified papillary thyroid microcarcinoma: post-operative management and treatment outcome in a single center

**DOI:** 10.1007/s12020-022-03060-5

**Published:** 2022-04-27

**Authors:** Wasit Kanokwongnuwat, Noppadol Larbcharoensub, Chutintorn Sriphrapradang, Chaiyawat Suppasilp, Kanungnij Thamnirat, Chaninart Sakulpisuti, Arpakorn Kositwattanarerk, Chirawat Utamakul, Chanika Sritara, Wichana Chamroonrat

**Affiliations:** 1 Division of Nuclear Medicine, Department of Diagnostic and Therapeutic Radiology, Faculty of Medicine Ramathibodi Hospital, Mahidol University, Bangkok, Thailand; 2 Division of Nuclear Medicine, Department of Radiology, Prapokklao Hospital, Chanthaburi, Thailand; 3 Department of Pathology, Faculty of Medicine Ramathibodi Hospital, Mahidol University, Bangkok, Thailand; 4 Division of Endocrinology and Metabolism, Department of Medicine, Faculty of Medicine Ramathibodi Hospital, Mahidol University, Bangkok, 10400 Thailand; 5 Department of Clinical Epidemiology and Biostatistics, Faculty of Medicine Ramathibodi Hospital, Mahidol University, Bangkok, Thailand

**Keywords:** Papillary thyroid microcarcinoma, Risk stratification, Post-operative management, Treatment outcome

## Abstract

**Purpose:**

This article aims to review and assess the post-operative management and treatment outcomes of papillary thyroid microcarcinoma (PTMC) in risk-stratified patients.

**Methods:**

We retrospectively analyzed the data of PTMC patients who underwent thyroid surgery with or without radioactive iodine treatment (RAI) in a single center between January 2011 and December 2017. Demographic and clinicopathologic data were collected. Risk stratification according to the 2015 American Thyroid Association guideline was applied.

**Results:**

Three hundred forty PTMC patients were included. Post-operative RAI was performed in 216/340 (63.53%) patients. In the non-RAI scenario, there were 122 low-risk and two intermediate-risk patients. In total, 261 (76.77%), 57 (16.76%), and 22 (6.47%) patients were classified as low, intermediate, and high risk, respectively. With a median follow-up time of 36 months (interquartile range: 23, 52), we found unfavorable outcomes (evidenced by imaging or out-of-range serum tumor marker levels: high thyroglobulin [Tg] or rising Tg antibody [TgAb] levels) in 8/340 (2.35%) patients, all of which received RAI. PTMC patients with unfavorable outcomes were stratified as low risk (4/261 [1.53%]), intermediate risk (1/57 [1.75%]), or high risk (3/22 [13.64%]). One death occurred in a patient with initial distant metastasis in the high-risk group. Initial high-risk stratification and initial stimulated Tg (of at least 10 ng/mL) were demonstrated as independent predictors for PTMC unfavorable outcomes (persistent or recurrent disease). Five patients with unfavorable outcomes (four with persistent disease and one with recurrent disease) had abnormal Tg or TgAb values despite unremarkable imaging findings. Moreover, 79/124 (63.71%) patients in the non-RAI scenario were only followed up with neck ultrasound.

**Conclusions:**

In general, at least 98% of low-risk and intermediate-risk PTMC patients showed favorable outcomes without persistent or recurrent disease, defined by either imaging or serum tumor markers. Nevertheless, aggressive disease could occur in few PTMC patients. Decisions on post-operative management and follow-up may be guided by initial high-risk stratification and initial stimulated Tg levels (≥10 ng/mL) as independent predictors for PTMC unfavorable outcomes. Monitoring using both imaging and serum tumor markers is crucial and should be implemented for patients with PTMC.

## Introduction

Patients with papillary thyroid microcarcinoma (PTMC) have tumors 1 cm or less in size, regardless of adverse features such as lymph node (LN) metastasis or distant metastasis [1]. The incidence of PTMC has increased in the last few decades. There were approximately 20,000 PTMC cases in the United States in the last 40 years [2] and PTMC accounts for about 20–30% of papillary thyroid cancer (PTC) [2, 3]. PTMC tumors are not always indolent [4–6] and PTMC-related mortality is ~0.5% [7, 8]. Risk stratification for differentiated thyroid cancer (DTC) patients is used as a prognostic tool to guide decisions on appropriate management that aim to achieve favorable outcomes. Treatment guidance in clinical practice is based on the 2015 American Thyroid Association (ATA) [1] and the modified version of the 2019 European Society of Medical Oncology (ESMO) guidelines [9]. Active surveillance without immediate surgery is acceptable for in a select group of low-risk PTMC patients, which usually includes asymptomatic [10] and elderly patients [11, 12]. Unilateral thyroid lobectomy (LT) is a treatment option for low-risk PTMC without additional lesions in the contralateral lobe [13–15]. Conversely, intermediate- or high-risk PTMC patients should receive bilobed thyroidectomy (BT) with or without radioactive iodine (RAI) treatment [16, 17]. For the evaluation of the response to treatment, both imaging and serum tumor markers (thyroglobulin [Tg] and thyroglobulin antibody [TgAb] levels) are combined and categorized [1, 9].

The purpose of this study is to review the post-operative management of PTMC and to assess the treatment outcome of PTMC in risk-stratified patients.

## Materials and methods

The study was approved by the Institutional Review Board (reference number: MURA2018/841). The institutional pathologic database was searched for results between January 2011 and December 2017. Patients with PTMC who underwent thyroid surgery during this period were collected.

Surgical pathology reports were reviewed and PTMCs were categorized as low risk, intermediate risk, or high risk based on the 2015 ATA risk stratification. A low-risk tumor was defined as an intrathyroidal PTMC with five or less LN metastases that were <0.2 cm in size at the greatest axis. An intermediate-risk tumor was defined as a tumor with aggressive histology (tall cell, hobnail variant, columnar cell carcinoma), microscopic extrathyroidal extension (ETE), vascular invasion, or LN metastases that were 0.2–3 cm in size. A high-risk tumor was defined as a gross ETE, incomplete tumor resection, distant metastases, and nodal metastases that were more than 3 cm in size. The data from pathology reports included tumor size, histologic variant, multifocality, presence of ETE, presence of lymphovascular invasion (LVI), presence of LN metastasis, presence of extranodal extension (ENE), and margin status. The involved or positive margin was considered as incomplete tumor resection. In the case of multifocality, the size of the largest tumor was recorded.

The data from patients’ medical records included age at diagnosis, sex, date and type of surgery, date and detail of RAI administration, initial and follow-up Tg and TgAb levels, presence of locoregional or distant metastasis at last visit, follow-up time, and mortality. Serum Tg and TgAb levels were evaluated by the Elecsys electrochemiluminescence immunoassay (Roche Diagnostic, USA). The general population ranges of the assays for Tg and TgAb were 3.5–77 ng/mL and 0–115 IU/mL, respectively.

Each patient had at least one follow-up visit at least 6 months after initial treatment with or without post-operative RAI, which included imaging or measurement of serum tumor marker levels. Tg and TgAb levels were measured as serum tumor markers. At the last follow-up, the outcome was determined as either favorable or unfavorable. Favorable outcome was defined as no evidence of disease on imaging, or serum tumor marker levels within the normal range. Favorable serum tumor marker levels were suppressed Tg <1 ng/mL or stimulated Tg <10 ng/mL and stable or falling TgAb levels. Conversely, the unfavorable outcome was defined as evidence of disease by imaging or by serum tumor marker levels out of the aforementioned range; patients with the unfavorable outcome were determined to have either persistent or recurrent disease. Persistent disease was considered as an unfavorable outcome at several visits until the last follow-up visit. Contrarily, patients were determined to have recurrent disease if they were classed with an unfavorable outcome at the last follow-up visit, despite classification with a favorable outcome during prior follow-up visits. During follow-up with thyroid hormone treatment, if any evidence of disease remained, TSH would be suppressed upon recommendation and clinical permissiveness.

The categorical variables are shown as frequency and percentage. The continuous variables are shown as median and interquartile range (IQR). The variables to predict unfavorable outcomes (either persistent or recurrent disease) were analyzed by univariate and multivariate logistic regression analyses. Variables with a *p* value < 0.25 in the univariate logistic regression analysis were included in the multivariate logistic regression analysis. A *p* value < 0.05 was considered statistically significant. Stata statistical software: Release 17 (College Station, TX: StataCorp LLC) was used for statistical analysis.

## Results

**Fig. 1:**
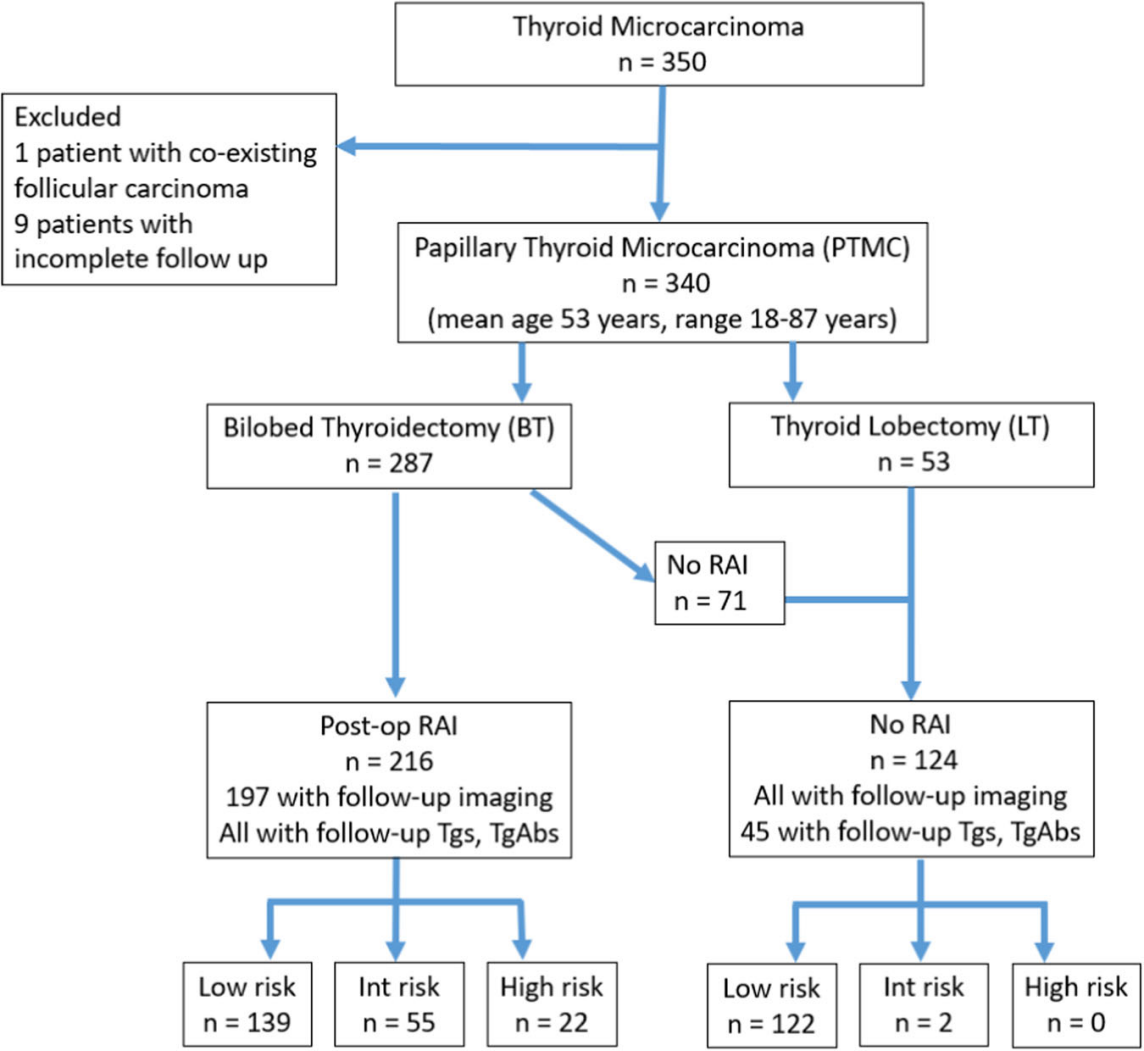
Study profile. BT bilobed thyroidectomy, Int intermediate, LT thyroid lobectomy, PTMC papillary thyroid microcarcinoma, RAI radioactive iodine, Tg thyroglobulin, TgAb thyroglobulin antibody

Three hundred fifty patients with PTMC were found in the institutional pathologic database. Nine patients with less than 6 months of follow-up data and one patient with coexisting follicular carcinoma were excluded (Fig. 1). Of the 340 PTMC patients included in this study, 287 patients (84.41%) underwent BT and 53 (15.59%) underwent LT. The mean patient age was 53 years (range: 18, 87 years), 86.76% of the patients were female, and 54.41% were younger than 55 years of age (Table 1).

**Table 1:** PTMC patient characteristics

Characteristics		Total 340 (100.00%)	Low risk 261 (76.77%)		Intermediate risk 57 (16.76%)		High risk 22 (6.47%)
			None 122 (46.74%)	RAI 139 (53.26%)	None 2 (3.51%)	RAI 55 (96.49%)	RAI 22 (100.00%)
Sex	Male	45 (13.24%)	14 (11.48%)	18 (12.95%)	1 (50.00%)	10 (18.18%)	2 (9.09%)
	Female	295 (86.76%)	108 (88.52%)	121 (87.05%)	1 (50.00%)	45 (81.82%)	20 (90.91%)
Age	<55 years	185 (54.41%)	67 (54.92%)	72 (51.80%)	0 (0.00%)	32 (58.18%)	14 (63.64%)
	≥55 years	155 (45.59%)	55 (45.08%)	67 (48.20%)	2 (100.00%)	23 (41.82%)	8 (36.36%)
Surgery	Bilobed thyroidectomy	287 (84.41%)	69 (56.56%)	139 (100.00%)	2 (100.00%)	55 (100.00%)	22 (100.00%)
	Thyroid lobectomy	53 (15.59%)	53 (43.44%)	0 (0.00%)	0 (0.00%)	0 (0.00%)	0 (0.00%)
Histological findings	Size, median (IQR), cm	0.5 (0.2, 0.8)	0.3 (0.2, 0.6)	0.5 (0.3, 0.8)	0.4 (0.1, 0.7)	0.7 (0.5, 0.9)	0.7 (0.6, 0.8)
	Multifocality	148 (43.53%)	21 (17.21%)	74 (53.24%)	1 (50.00%)	37 (67.27%)	15 (68.18%)
	Aggressive histology^a^	1 (0.29%)	0 (0.00%)	0 (0.00%)	0 (0.00%)	1 (1.82%)	0 (0.00%)
	Microscopic extrathyroidal extension	35 (10.29%)	0 (0.00%)	0 (0.00%)	1 (50.00%)	22 (40.00%)	12 (54.55%)
	Lymphovascular invasion	18 (5.29%)	0 (0.00%)	0 (0.00%)	0 (0.00%)	14 (25.45%)	4 (18.18%)
	Positive margin	17 (5.00%)	0 (0.00%)	0 (0.00%)	0 (0.00%)	0 (0.00%)	17 (77.27%)
	Lymph node metastasis	34 (10.00%)	0 (0.00%)	0 (0.00%)	1 (50.00%)	23 (41.82%)	10 (45.45%)
	Extranodal extension	4 (1.18%)	0 (0.00%)	0 (0.00%)	0 (0.00%)	0 (0.00%)	4 (18.18%)
Initial RAI findings	Lymph node metastasis	25 (7.35%)	0 (0.00%)	0 (0.00%)	0 (0.00%)	22 (40.00%)	3 (13.64%)
	Distant metastasis	1 (0.29%)	0 (0.00%)	0 (0.00%)	0 (0.00%)	0 (0.00%)	1 (4.55%)
Initial stimulated Tg, median (IQR), ng/mL	Overall	1.71 (0.32, 6.50)	NA	1.42 (0.20, 4.34)	NA	2.90 (0.50, 8.70)	3.66 (0.69, 14.20)
	Excluded elevated TgAb	2.84 (0.90, 8.97)	NA	2.50 (0.65, 7.18)	NA	5.08 (1.7, 11.1)	5.87 (1.10, 18.50)
Initial TNM staging	Stage I	320 (94.12%)	122 (100.00%)	139 (100.00%)	1 (50.00%)	40 (72.73%)	18 (81.82%)
	Stage II	20 (5.88%)	0 (0.00%)	0 (0.00%)	1 (50.00%)	15 (27.27%)	4 (18.18%)
Unfavorable outcome		8 (2.35%)	4 (1.53%)		1 (1.75%)		3 (13.64%)

All values are *n* (%) unless otherwise stated

*IQR* interquartile range, *NA* not applicable, *RAI* radioactive iodine, *Tg* thyroglobulin, *TgAb* thyroglobulin antibody, *TNM* tumor node metastasis

^a^Tall cell variant

Histological findings revealed that the median tumor size was 0.5 cm (IQR: 0.2, 0.8) and 43.53% of patients (148/340) indicated multifocality. One patient (0.29%) with aggressive histology (tall cell variant) was noted. Microscopic ETE was found in 35 patients (10.29%) without gross ETE. LVI and positive margin were found in 18 (5.29%) and 17 (5.00%) patients, respectively. All patients with LT showed free margin status. Histological LN metastasis was revealed in 34 patients (10.00%), four of which had ENE. In addition, RAI-avid LN metastasis was observed in 25 patients, eight of whom already had histologically proven LN metastasis.

According to the 2015 ATA risk stratification, the initial risk stratification was categorized using initial histology and imaging findings into low-risk, intermediate-risk, and high-risk groups, which included 261 (76.77%), 57 (16.76%), and 22 (6.47%) patients, respectively. The common histological features in the intermediate-risk group were LN metastasis (42.11% [*n* = 24]) and microscopic ETE (40.35% [*n* = 23]). The most common feature in the high-risk group was positive margin (77.27% [*n* = 17]).

According to the American Joint Committee on Cancer (AJCC) 8th edition TNM staging (classification for Tumor, Node, and Metastatic spread), the initial staging identified 320 (94.12%) stage I patients and 20 (5.88%) stage II patients.

Following all initial treatments, the median follow-up time was 36 months (IQR: 23, 52). Only ten patients had a follow-up time of less than 12 months. At the last follow-up, all 124 patients in the non-RAI scenario had a favorable outcome with no evidence of persistent or recurrent disease. Although neck ultrasound was documented for each non-RAI patient during a follow-up visit, 79 (63.71%) patients had no available serum tumor marker levels, including 53 patients with LT.

Post-operative RAI treatment at 1.1–5.5 GBq (30–150 mCi) was given within 3 months after surgery in 216 (63.53%) patients. Of all post-operative RAI patients, 117 patients received RAI at 1.1 GBq (30 mCi). Nineteen (8.80%) patients had no follow-up imaging except for a post-operative RAI treatment scan; however, all of these patients had follow-up Tg and TgAb analyses.

Overall, 332 (97.65%) patients had favorable outcomes, whereas 8 (2.35%) patients showed unfavorable outcomes. PTMC patients with unfavorable outcomes were proportionally stratified into low-risk (4/261 [1.53%]), intermediate-risk (1/57 [1.75%]), and high-risk (3/22 [13.64%]) groups. Of the eight patients with unfavorable outcomes, five (four persistent and one recurrent disease) had out-of-range tumor marker levels without evidence of disease on imaging (Table 2).

**Table 2:** Patients with unfavorable outcome at last follow-up

No	Risk	Age (years)	Sex	Tumor size (cm)	Multifocality	mETE	LVI	Margin	LN size (cm)	ENE	Initial distant metastasis	Initial stimulated Tg (ng/mL)	Initial TgAb (IU/mL)	F/U (months)	Final suppressed Tg (ng/mL)	Final TgAb (IU/mL)	Unfavorable outcome	Imaging
1	Low	55	F	0.8	+	−	−	−	−	−	−	16.80	<10.00	44	1.15	<10.00	Persistent	Unremarkable neck ultrasound
2	Low	66	F	0.1	−	−	−	−	−	−	−	3.00	204.00	50	11.50	<10.00	Persistent	Unremarkable neck ultrasound
3	Low	68	F	0.2	−	−	−	−	−	−	−	14.53	<10.00	36	128.00	<10.00	Recurrent	Lung metastasis on chest CT
4	Low	78	F	1	−	−	−	−	−	−	−	0.20	41.70	54	<0.04	775.00	Recurrent	Unremarkable neck ultrasound and RAI scan
5	Intermediate	35	F	0.8	+	−	−	−	1.2	−	−	190.60	11.70	65	3.12	<10.00	Persistent	Unremarkable neck+chest CT and RAI scan
6	High	40	M	0.6	+	+	+	−	3	−	+	50,000.00	698.80	38	42,601.00	248.10	Persistent with death	Lung and bone metastasis on RAI scans
7	High	46	F	0.7	−	−	+	+	1	+	−	234.60	<10.00	24	4.65	<10.00	Persistent	Unremarkable neck ultrasound
8	High	59	F	0.4	−	−	−	−	6	−	−	2.50	>4000.00	46	<0.04	>4,000.00	Recurrent	Nodal metastasis on neck ultrasound

*CT* computed tomography, *ENE* extranodal extension, *F/U* follow-up, *LN* lymph node, *LVI* lymphovascular invasion, *mETE* microscopic extrathyroidal extension, *Tg* thyroglobulin, *TgAb* thyroglobulin antibody

− Denotes absence and + denotes presence

Of three unfavorable outcome patients with proven imaging, one had recurrent nodal metastasis and two had distant metastases (one persistent and one recurrent). One death was noted in a male patient in whom distant metastasis was found in the initial analysis. This patient was in the high-risk PTMC group and showed initial extensive nodal, lung, and bone metastases. This patient also had human immunodeficiency virus and was therefore immunocompromised.

The univariate analyses showed that LVI, LN metastasis, ENE, initial stimulated Tg of at least 10 ng/mL, initial high-risk group classification, and initial TNM staging were significant prognostic factors for unfavorable outcomes (Table 3). In the multivariate logistic regression analysis, only two factors remained significant: initial stimulated Tg of at least 10 ng/mL and initial high-risk group classification (Table 4). In addition, initial stimulated Tg of at least 10 ng/mL could improve the model compared with using high-risk group classification alone (Likelihood Ratio Chi-squared statistic of 7.01, *p* = 0.008). Unfortunately, only 214 patients exhibited initial stimulated Tg; thus, the multivariate analysis could be considered as a subgroup analysis of the cohort. The interaction effect between initially high stimulated Tg and initial high-risk group classification was evaluated; however, no apparent effect was found.

**Table 3:** PTMC factors associated with unfavorable outcomes at last follow-up (univariate logistic regression analyses)

Variables		Patients (*n* = 8) with unfavorable outcome n/n (%)	Univariate analysis OR (95% CI)	*p* value
Sex	Male	1/45 (2.22%)	1.07 (0.13, 8.90)	0.950
	Female	7/295 (2.37%)	1	
Age	≥55 y	5/155 (3.23%)	2.02 (0.48, 8.60)	0.340
	<55 y	3/185 (1.62%)	1	
Histologic findings				
Size (*n* = 339)^a^	≥0.5 cm	5/179 (2.79%)	1.50 (0.35, 6.40)	0.581
	<0.5 cm	3/160 (1.88%)	1	
Multifocality	Present	3/148 (2.03%)	0.77 (0.18, 3.29)	0.728
	Absent	5/192 (2.60%)	1	
Microscopic extrathyroidal extension	Present	1/35 (2.86%)	1.25 (0.15, 10.48)	0.836
	Absent	7/305 (2.30%)	1	
Lymphovascular invasion	Present	2/18 (11.11%)	6.58 (1.23, 35.23)	0.028*
	Absent	6/322 (1.86%)	1	
Initial lymph node metastasis (histology and imaging)	Present	4/51 (7.84%)	6.06 (1.47, 25.08)	0.013*
	Absent	4/289 (1.38%)	1	
Extranodal extension	Present	1/4 (25.00%)	15.67 (1.44, 169.92)	0.024*
	Absent	7/336 (2.08%)	1	
Positive margin	Present	1/17 (5.88%)	2.82 (0.33, 24.33)	0.345
	Absent	7/323 (2.17%)	1	
Laboratory				
Initial stimulated Tg (*n* = 214)	≥10 ng/mL	5/40 (12.50%)	8.14 (1.86, 35.66)	0.005*
	<10 ng/mL	3/174 (1.72%)	1	
Staging and risk stratification				
Initial risk stratification	Non-high	5/318 (1.57%)	1	0.003*
	High	3/22 (13.64%)	9.88 (2.20, 44.49)	
Initial TNM staging	Stage 1	6/320 (1.88%)	1	0.039*
	Stage 2	2/20 (10.00%)	5.81 (1.10, 30.87)	

*CI* confidence interval, *OR* odds ratio, *Tg* thyroglobulin, *TNM* tumor node metastasis

*p value < 0.05

^a^In one patient, histology indicated a tumor size of “<1 cm”

**Table 4:** Multivariate logistic regression analysis of unfavorable outcome at last follow-up among patients who had initial stimulated Tg (*n* = 214)

Variables (*n* = 214)		Adjusted OR (95% CI)	*p* value
Initial risk stratification	Non-high	1	0.042*
	High	5.18 (1.06, 25.25)	
Initial stimulated Tg	≥10 ng/mL	7.53 (1.67, 33.91)	0.009*
	<10 ng/mL	1	

*CI* confidence interval, *OR* odd ratio, *Tg* thyroglobulin

*p value < 0.05

## Discussion

Depending on risk assessment, several management options are available for PTMC patients. Active surveillance without immediate surgery is acceptable for a select group of low-risk PTMC patients [18] that are asymptomatic [10] and elderly [11, 12]. Active surveillance is less costly and is associated with less adverse effects, including permanent effects such as vocal cord paralysis and hypoparathyroidism, which can occur with thyroid surgery [19]. LT is an option for low-risk PTMC without additional lesions in the contralateral lobe [13–15]. Conversely, PTMC patients with intermediate- or high-risk classification should be treated with BT with or without RAI [16, 20]. PTMC patients with multifocality and large tumor size are often referred for total thyroidectomy [21].

In our study, 53/340 (15.59%) patients underwent LT; of these, none received post-operative RAI and none showed involved margin status. Six out of these 53 patients had multifocality in the resected unilateral thyroid lobe. No contralateral lobe lesion was founded during follow-up. In Table 1, patients classed as high risk were more likely to receive post-operative RAI. Specifically, post-operative RAI was performed for 139/261 (53.26%) low-risk, 55/57 (96.49%) intermediate-risk, and 22/22 (100%) high-risk patient groups. These results imply that risk-stratified features were considered in the post-operative RAI decision. Even within the low-risk group, our analysis showed that post-operative RAI patients had larger tumor sizes and were more likely to have multifocality than non-RAI receivers.

Post-operative or adjuvant RAI showed long-term benefit for high and intermediate-risk DTC [22, 23], including some PTMC [24–26]. The benefit of adjuvant RAI in low-risk DTC patients was debatable [27–29] until recent publication of the prospective, randomized controlled trial on low-risk DTC patients with or without RAI administration and following the occurrence of functional, structural and biologic events [30]. Leboulleux et al. [30] found that the non-RAI group was non-inferior to RAI-received group at 3 years follow-up. Currently, there is a general consensus to avoid RAI in intra-thyroid PTMC; the risk of recurrence is minimal that it can hardly been reduced. Conversely, more aggressive PTMC may have post-operative RAI with similar indication of aggressive DTC. However, either more or less aggressive PTMC should have follow-up with both tumor markers and imaging, accordingly.

Although PTMC nodal metastasis varied between 32 and 57% [31–33] at the initial thyroid surgery, we found cervical nodal metastasis in 10% of patients after surgery. However, metastasis identification increased to 15% in patients with RAI administration and imaging, and none of these patients were in the low-risk group. Mourao et al. suggested that RAI may not be necessary for patients with low (<0.3 ng/mL) post-operative suppressed Tg, non-elevated TgAb, and negative neck ultrasound findings [34]. Despite this, in our study, all the PTMC patients with pathologically proven cervical nodal metastasis received post-operative RAI, except one. An intermediate-risk 55-year-old male patient without post-operative RAI initially underwent total parathyroidectomy because of secondary hyperparathyroidism; this patient had end-stage renal disease and ongoing hemodialysis. One “parathyroid” lesion was later identified as a 0.6-cm LN with metastatic PTC. In the following months, the patient had total thyroidectomy (multifocal PTMC up to 0.1 cm) and the remaining portion of the hyperactive parathyroid gland was also resected. Another intermediate-risk 65-year-old female patient without post-operative RAI in our study had only microscopic ETE without aggressive histology or LN metastasis. Neither of the intermediate-risk, non-RAI patients discussed here had evidence of disease recurrence on imaging and did not have high Tg or TgAb levels during follow-up visits.

Of 261 low-risk patients, 139 (53.26%) received post-operative RAI. Four of these had unfavorable outcomes: two with persistent and two with recurrent disease at the last follow-up. In contrast, we found no evidence of disease in 124 non-RAI patients, including 122 low-risk and two intermediate-risk patients. Surprisingly, 79 (63.71%) non-RAI patients were only followed up with neck ultrasound, and Tg and TbAb levels were not measured during visits. At the time, no appropriate Tg threshold was indicated in any guidelines for patients with partial thyroidectomy, lobectomy, or without RAI ablation; therefore, obtaining Tg and TgAb measurements may have been difficult because of issues related to patient acceptance, especially for patients with non-uniform Tg and TgAb values. In 45/122 (36.29%) non-RAI patients with available follow-up Tg and TgAb analyses, the mean suppressed Tg level was 0.19 ng/mL (range: 0.04, 0.91 ng/mL). Three of these patients had elevated but non-rising TgAb levels.

Torlontano et al. suggested that neck ultrasound should be used as a primary tool during the follow-up of PTMC patients without post-operative RAI [35]. Moreover, response to treatment, measured by follow-up Tg and TgAb levels, in DTC patients without post-operative RAI for either LT or BT were mentioned [9, 36, 37]. Regardless of previous actions, monitoring with both imaging and serum tumor markers could be useful. Using only these methods, evidence of disease as the biochemical unfavorable outcome could be detected in our study (Table 2; patient no. 1, 2, 4, 5, 7).

In this study, we regarded histologic positive margins as incomplete tumor resection; therefore, all patients with histologic positive margins were classified as high risk. Of 17 patients with a positive margin, only 1 patient, a 40-year-old female, had an unfavorable outcome. This patient had a positive margin as well as nodal metastasis with ENE. Raruenrom et al. found that a microscopic positive margin in DTC was associated with incomplete response after RAI at a median follow-up of ~10 months [38]. However, Wang et al. retrospectively reviewed 3,664 patients with DTC and reported that the microscopic positive margin was not an independent predictor of local recurrence at a median follow-up of 50 months [39]. In our study, which included a median follow-up time of 36 months, the 16 patients with positive margins did not have recurrent disease.

Markantes et al. suggested the optimal values of pre-RAI stimulating Tg levels and non-elevated TgAb levels, when used as predictive factors for persistent and recurrent DTC disease, were 12.75 and 8.05 ng/mL, respectively [40]. Of eight patients with unfavorable outcomes in our study, five had non-elevated TgAb levels based on the non-elevated TgAb threshold of 115 IU/mL. Of these, four patients had initial stimulated Tg levels of at least 14.5 ng/mL, three of these patients had persistent disease (Table 2; patient no. 1, 5, 7), and one (patient no. 3) had recurrent disease of new lung metastasis. However, one patient (no. 4) with biochemical recurrence (rising TgAb levels) had initial stimulated Tg levels of only 0.2 ng/mL and initial non-elevated TgAb levels of 41.7 IU/mL. Of 261 patients with available follow-up Tg and TgAb analyses, 15 (5.75%) patients had elevated TgAb levels. Amazingly, 3/8 (37.50%) patients with unfavorable outcome (Table 2: patient no. 4, 6, 8) had elevated TgAb levels. Only patient no. 4 with rising TgAb levels had no confirmed imaging of recurrence. Further follow-up may ascertain structure recurrence in the patient. The significance of elevated serum Tg or TgAb remains indefinite until it shows its rising trend over time or proven structural disease.

Previous studies have identified several independent factors associated with PTMC unfavorable consequences. Jeon et al. revealed ENE as a predictive factor for distant metastasis [31]. Mercante et al. determined that ETE, capsular invasion, and nodal metastasis at initial presentation were factors affecting cervical LN recurrence and distant metastasis [41]. Siddiqui et al. demonstrated that age, ETE, and multifocality were factors correlated with local recurrence [42]. All of these factors were histologic features. Our results indicate that laboratory findings (initial stimulated Tg ≥10 ng/mL) and risk stratification (initial high-risk group) are independent predictors for PTMC unfavorable outcomes (persistent or recurrent disease).

The main limitations of our study were the retrospective design and the relatively short follow-up of 3 years. We may have identified additional unfavorable outcomes or increased PTMC recurrence with longer follow-up times [43].

In conclusion, at least 98% of low-risk and intermediate-risk PTMC patients showed favorable outcomes without persistent or recurrent disease on imaging or based on serum tumor markers. Nevertheless, aggressive disease could occur in few PTMC patients. Patients presenting with factors associated with persistent or recurrent disease, as determined in our study, will require additional attention during follow-up. Serial monitoring with both imaging and serum tumor markers is crucial and should be implemented for PTMC patients.
